# Factors associated with the use of probiotics in patients with inflammatory bowel disease

**DOI:** 10.12688/f1000research.2-69.v1

**Published:** 2013-03-04

**Authors:** Claire Louise Agathou, Ian LP Beales

**Affiliations:** 1 Norwich Medical School, University of East Anglia, Norwich, NR4 7TJ, UK; 2 Department of Gastroenterology, Norfolk and Norwich University Hospitals NHS Foundation Trust, Norwich, NR4 7UZ, UK

## Abstract

**Background: **Probiotic preparations are heavily promoted in the United Kingdom and are widely available to purchase. Probiotics have multiple effects on gastrointestinal functions and may have beneficial or even harmful effects in inflammatory bowel disease (IBD). Various complementary and alternative medicines are commonly used by IBD patients but there is much less data specifically on the use of probiotics.

**Aim:** To examine the current use of probiotics by IBD patients and determine the factors associated with probiotic use.

**Methods: **Subjects with IBD undergoing routine care at a UK teaching hospital underwent a standardized structured questionnaire-interview. Current use of probiotics was explored and patient- and disease-related factors examined. IBD-related quality of life was assessed with the short inflammatory bowel disease questionnaire (S-IBDQ). Logistical regression was used to explore factors associated with probiotic use.

**Results: **Forty subjects were interviewed.  Probiotic use was common, 40% of subjects being regular users. Probiotic use was significantly associated with a shorter duration of IBD since diagnosis, a diagnosis of Crohn’s disease, formal post-18 education and lower quality of life as assessed by the S-IBDQ. A preference for the taste of the preparation was as common a reason for using probiotics as were potential disease modifying effects. Non-users reported that the costs of the preparations and doubts about efficacy were the primary reasons for non-use.

**Conclusions: **In this study probiotic use was common in IBD patients. Several patient- and disease- related factors, including a lower perceived quality of life, were associated with the use of probiotics.

## Introduction

The use of probiotics within the field of Gastroenterology is an area of significant current interest. The gut is home to millions of microorganisms and collectively this is often referred to as the “ *gut microbiome*” ^1^. This refers to coexistence of beneficial and pathological microorganisms within the gut flora, which under usual physiological “ *healthy*” states are considered normal. *Clostridium difficile* ( *C. difficile*) demonstrates this balance – many normal individuals carry this organism within their large bowel and yet exhibit no associated symptoms; however, when the more beneficial organisms are reduced, for example by use of antibiotics, *C. difficile* dominates the GI tract resulting in an acute diarrhoeal-type disease ^2^. The prospect that we may be able to improve the natural history of bowel diseases through manipulation of the normal gut homeostasis between various health-promoting and health-endangering microorganisms is the basis of potential for the role of probiotics in health and disease ^3– 5^. Probiotics are defined as live microbial supplements which exert a beneficial effect on health and are non-pathogenic or toxic ^6^.

Recent evidence suggests that the presence of different bacterial species in the colon can have a significant impact on the immune functioning of the gastrointestinal tract and that manipulation of normal gut microbiome homeostasis can alter local immunity within the luminal gut as well as systemically ^3, 4, 7, 8^. Idiopathic chronic inflammatory bowel diseases (IBD) such as Crohn’s disease and ulcerative colitis are characterised by persistent or episodic inflammation of the gastrointestinal mucosa and it appears that these patients are mounting overwhelming immune responses to non-pathogenic gut bacteria which would otherwise be ignored in the gut of a healthy host ^9^. The gut microbiome differs significantly between healthy controls and IBD patients, particularly within the inflammatory colonic lesions, which are found to contain greater numbers of unfavourable bacteria ^7, 9^. Luminal colonic flora and the immunological response of the gut play a major role in initiation and perpetuation of chronic IBD ^7, 8, 10^, although whether these alterations in gut microbiome are primary causes of the diseases or secondary phenomena resulting from the disease currently remains undecided.

Probiotics are non-pathogenic, live microbial supplements which when taken on a regular basis, claim to offer an immune advantage by increasing the balance of health-promoting bacteria such as lactobacilli. Prolonged exposure of supplemental lactobacilli induces their translocation and adherence to human intestinal epithelial cells which are capable of activating macrophages ^9^. Enhancing the immunomodulatory effect of intestinal flora to inflammation by such means is thus of great current interest for patients with IBDs yet studies supporting this theory are limited.

The most commonly used bacterial micro-organisms are bifidobacterium and lactobacillus and marketing strategies promote these heavily on the basis that they will improve health and be of benefit to the gastrointestinal tract. For the past decade, probiotics have been marketed as food supplements, most commonly in the form of drinks or tablets, widely available without prescription from supermarkets or from internet sites. Thus, there is uncertainty about the prevalence of use within the general population and in particular amongst patients who suffer with inflammatory bowel conditions. We have hypothesized that these preparations would be especially attractive to patients with chronic IBDs: these conditions may require continued therapy with powerful immunosuppressive drugs and the concept of restoring the balance of bacteria in the gut with a food supplement is likely to be attractive to many people. However we do not know how many people with IBD are using these preparations. Although widely regarded as completely safe and natural, it is even possible that probiotics could be harmful: the powerful immunosuppressive drugs taken by many patients with IBD or the underlying disease could alter the response to these otherwise harmless bacteria, and rare cases of invasive systemic disease have been reported with probiotics ^6^. It is now clear that many foodstuffs and dietary supplements can interact with prescription drugs and either increase or decrease the effect of these drugs ^10, 11^; other theoretical problems with probiotics in IBD include the possible transmission of bacterial antibiotic resistance from non-pathogenic to pathogenic bacteria and the generation of as yet unreported negative effects upon the gastrointestinal immune system ^6^. Also probiotics have been shown to accelerate gastrointestinal transit and could induce diarrhoea or a change in stool frequency in an IBD patient that might otherwise be assumed to be a flare of active disease ^10, 12^.

Despite the potential benefits or adverse effects of probiotics in IBD as outlined above, we do not currently know how prevalent the use of these supplements are. Neither do we know why certain patients may be taking them. Therefore it is important to determine how commonly these supplements are used in IBD and how interested IBD patients are in taking them. Furthermore, determining which are the main drivers of probiotic usage may shed light on issues with the current delivery of care. Probiotic uses may reflect a dissatisfaction with current therapies or demonstrate a positive interest in more natural therapies; thus we may be able to identify areas of need within our service and hence provide additional resources and support for patients. Despite the widespread availability of probiotics, there are relatively few studies examining the use of these preparations in the IBD population ^13– 17^, and in particular there is a paucity of information related to patients in the United Kingdom.

We wherefore aimed to determine the prevalence of probiotic use amongst patients with IBD and assess which disease-related and demographic factors are influencing their consumption. As probiotics are relatively new “therapies”, often classified as complementary alternative medication (CAM), it is important to determine variables that may influence their usage as these may create bias in later studies seeking to quantify their overall effects.

## Methods and materials

### Inclusion and exclusion criteria

Study subjects were selected from IBD outpatient clinics and inpatients under the care of the Adult Gastroenterology Department of the Norfolk and Norwich University Hospital. Consecutive adult (> 18 years old) patients with a confirmed diagnosis of Crohn’s disease or ulcerative colitis, from sessions when the student researcher was available, were eligible for recruitment.

Patients requiring enteral nutrition were excluded as oral intake was dictated by their prescription and not personal choice. Patients with indeterminate colitis were excluded to aid classification of the results. Subjects unable to complete the interview questionnaire in English were excluded. All participants gave informed consent and the study was approved by the Norfolk and Norwich University Hospital research governance committee and Cambridge 3 Research Ethics committee.

### Interview questionnaire

All subjects underwent a structured interview and completion of a structured questionnaire, (see Appendix 1) administered by the same trained student-researcher (CLA). Questions were separated into sociodemographic and disease-related variables including an assessment of the patient’s health-related quality of life by incorporating the S-IBDQ (Short Inflammatory Bowel Disease Questionnaire), a validated screening tool for patients with IBD ^13^.

### Study size and analysis

The study was designed as an exploratory study aiming to test the feasibility and acceptability of the questionnaire and methodology in the IBD population and provide estimates on the prevalence of probiotic use in the IBD population, which could be possibly used to inform the design of a subsequent larger study. For this study probiotic use was defined as regularly using at least one single probiotic preparation per week for at least one month. For the purposes of analysis, age was split into 2 groups, younger (18–55) and older (56+). SPSS 19.0 was used for statistical analysis: Fisher’s Exact Test was used to examine categorical data on age, duration of diagnosis and education level and Mann-Whitney U-test was used to examine the results of the S-IBDQ. Unconditional logistic regression was used to calculate odds ratios (OR) with 95% confidence intervals (CI) for the use of probiotics adjusted for age, gender, IBD type, duration of diagnosis and educational status.

## Results

40 patients were recruited between October 2010 and February 2011, 8 inpatients and 32 outpatients. Subjects were evenly split between Crohn’s Disease (20) and ulcerative colitis (20), and the overall age range was 18–78 (median age range 51–55, mode age range 61–65). Thirteen subjects were male and 27 female. Sixteen patients (40%) were considered to be regular users of probiotics (that is, using probiotics at least once per week). Crude data are appended in Appendix 2.

Probiotic use was much commoner in subjects with Crohn’s disease (13/20) than in those with ulcerative colitis (3/20) (odds ratio (OR) 9.8, 95% confidence interval 2.20 – 54.9) (p < 0.01).

The results describing probiotic use with different sociodemographic variables are shown in Table 1. Men used probiotics less commonly than women, but this was not statistically significant. Similarly, probiotic use was non-significantly commoner in the younger age group. However, probiotic use was significantly more common in those subjects who had continued in formal education after the age of 18 (OR 8.15, 95% CI 1.8 – 44.9) (p < 0.01).

**Table 1.** Demographic variables of probiotic users vs. non-users.

		Frequency (%)			p-value
**Probiotic use**		**No** **n = 24**	**Yes** **n = 16**	**Total** **n = 40**	
**Gender**	Male Female	10 (41.7) 14 (58.3)	3 (18.8) 13 (81.3)	13 (32.5) 27 (67.5)	0.177
**Age group**	18–55 years 56 years +	10 (41.7) 14 (58.3)	12 (75.0) 4 (25.0)	22 (55.0) 18 (45.0)	0.054
**Education**	High school College/University	16 (66.7) 8 (33.3)	3 (18.8) 13 (81.3)	19 (47.5) 21 (52.5)	0.004

**Figure 1.**
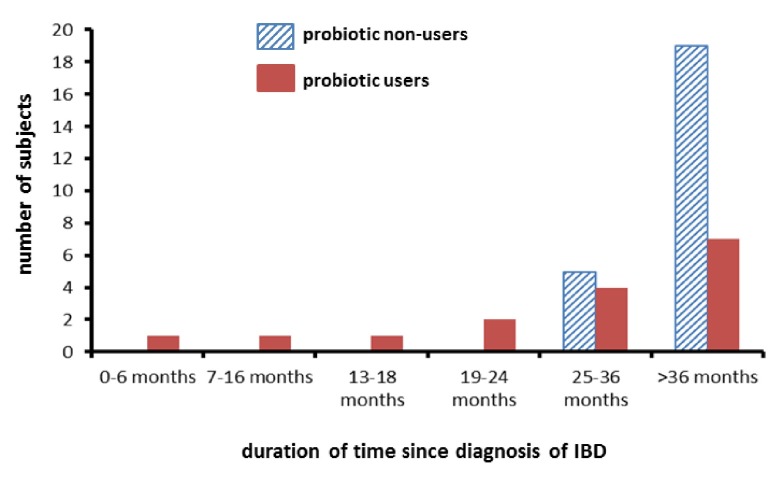
Distribution of time since diagnosis of IBD in probiotic users and non-users.

Disease-related factors are shown in Table 2. Within our sample there appeared to be a different distribution of disease duration (time since the diagnosis of IBD) between probiotic users and non-users ( Figure 1). There was a greater variability in disease duration since diagnosis in users than in non-users. When probiotic use was examined by duration of disease, probiotic use was commoner in those with a shorter duration of disease (< 36 months) (OR 9.83, 95% CI 1.19 – 258.3) (p < 0.05).

**Table 2.** Disease-related variables of probiotic users vs. non-users.

		Frequency (%)		Total	p-value
**Probiotic use**		**No** **n = 24**	**Yes** **n = 16**	**Total** **n = 40**	
**IBD type**	Crohn’s disease Ulcerative colitis Total	7 (29.2) 17 (70.8) 24	13 (81.3) 3 (18.8) 16	20 (50.0) 20 (50.0) 40	0.003
**Duration of disease**	Less than 36 months 36 months + Total	5 (20.8) 19 (79.2) 24	9 (56.3) 7 (43.8) 16	14 (35.0) 26 (65.0) 40	0.041

Logistic regression was used to adjust odds ratios for age, sex, duration of disease and educational attainment. The results are shown in Table 3. After adjustment, probiotic use was found to be significantly associated with Crohn’s disease compared to ulcerative colitis, shorter duration of disease and higher educational attainment.

**Table 3.** Adjusted odds ratios for probiotic usage.

		Odds Ratio (OR)	95% CI	p-value
**Gender**	Male Female	0.405 1.00	(0.05–3.67)	0.412
**Age**	18–55 years 56 years +	1.200 1.00	(0.13–10.87)	0.869
**IBD type**	Crohn’s disease Ulcerative colitis	30.882 1.00	(2.252–423.27)	0.009
**Duration of disease**	3 years or less 3 years +	17.270 1.00	(1.12–265.87)	0.037
**Education**	High school or less Higher education (College/Uni)	0.093 1.00	(0.01–0.92)	0.038

Data from the S-IBDQ were used to analyze the relationship between IBD symptoms and probiotic use. As shown in Table 4, probiotic use was significantly associated with overall poorer perceived health-related quality of life. However on comparison of each individual domain, there appears to be no significant difference in S-IBDQ scores in both systemic and bowel-specific outcomes. Non-users scored more favourably in both emotional and social domains and these were both found to show statistical significance. Within our population, scores from the S-IBDQ did not correlate with other sociodemographic or disease related factors.

**Table 4.** S-IBDQ outcomes: probiotic users vs. non-users.

		Median score (interquartile range)		p-value
*Probiotic use*		*Non-users*	*Users*	
**S-IBDQ domains**	Systemic Emotional Social Bowel	16.50 (4.00) 16.00 (6.00) 11.00 (4.00) 11.50 (4.00)	16.00 (3.00) 13.00 (5.00) 9.00 (5.00) 10.50 (5.00)	0.102 0.049 0.034 0.116
	Total	56.50 (12.75)	47.00 (16.25)	0.018

*(The lower the score, the poorer the health related QOL of the patient)*

We also explored the reasons subjects gave for using or non-using probiotics, and the results are shown in Table 5. Although the numbers in each group were small, there were some potentially different reasons that seemed to drive probiotic use. In the non-users, the relatively high costs and a perception that the probiotics would not significantly improve symptoms were common reasons given; interestingly a substantial minority group (8 subjects) said they avoided probiotics because of perceived lactose intolerance and the possibility that milk-containing products would make the symptoms worse. Half of probiotic users gave the taste of probiotic preparations as their primary reason for using them; the other half suggested that they used probiotics primarily to improve their IBD symptoms in some way.

**Table 5.** Primary reasons for using or not using probiotics.

	Probiotic users (n = 16)	Probiotic non-users (n = 24)
Preferred taste	8 (50%)	
Symptom prevention	1 (6.25%)	
Symptom reduction	2 (12.5%)	
Symptom reduction and prevention	5 (31.25%)	
Will not prevent symptoms		1 (4.2%)
Will not reduce symptoms		1 (4.2%)
Will not prevent or reduce symptoms		5 (20.8%)
Cost		9 (37.5%)
Others		8 (33.3%) (all 8 concerned that lactose-containing products would exacerbate symptoms)

## Discussion

Although our study is relatively small, we have found that probiotic use in the IBD population is associated with Crohn’s disease rather than ulcerative colitis, shorter disease duration since diagnosis, higher educational attainment and a poorer perceived quality of life. All probiotics were purchased, none were obtained on prescription. The majority of probiotic use was in the form of yoghurts or drinks, although one patient with ulcerative colitis regularly used the specific probiotic preparation VSL #3 (a concentrated probiotic food supplement preparation, Ferring, West Drayton, UK), which he purchased via the internet. Interestingly probiotic use was common in the IBD population, despite the lack of evidence supporting the use of these drinks and yoghurts; this probably reflects popular interest in healthy lifestyles and non-drug therapies. Proprietary probiotic drinks have had significant marketing through television advertisements and many users said they were inclined to purchase brands which were being actively promoted. Interestingly, over one third of non-users stated that their primary reason for not using probiotics in their diet was on account of cost, thus indicating an important reason which may deter patients from purchasing them, particularly as their effects are considered beneficial only with long-term, continuous use.

Within our total population, level of education appeared to have a strongly significant association with probiotic use: over 80% of the participants who claimed to use probiotics had also continued to higher education. This again may be related to the cost of probiotics which is deflecting participants on lower salaries (higher education being one determinant of earning power). Increased probiotic use in those continuing with post-18 education may also reflect individual awareness or social awareness about probiotics.

The commonest reason provided by users regarding their primary choice to consume probiotics was that they liked the taste. This was perhaps slightly surprising, as we may have expected disease-related issues to be the primary drivers. In light of this it is interesting that none of the non-users gave taste as a primary reason for not using probiotics. Within both groups, reasons governing the decision to either use or not use probiotics were taste preference (users) or high cost (non-users), respectively – this reflects that probiotics may be considered more as a luxury/food item, rather than a medical therapy. In the current study we did not explore experiences with previous use of probiotics.

Subjects with Crohn’s disease were more commonly users of probiotics than those with ulcerative colitis and this did not seem to be related to any specific factors and was not obviously related to perceived quality of life. At present, the numbers in the study are too small to provide any further data on previous surgeries or drug exposures as potential drivers to the use of probiotics. Within the free text of the questionnaire, of the non-users, 7 ulcerative colitis patients commented that they avoided the use of probiotics as they considered them to be similar to milk and, as they avoid all dairy produce for symptom prevention, this ruled out any desire or option to use them. Only one patient with Crohn’s disease stated that she avoided probiotics for similar reasons. If probiotic supplementation does prove in future to offer a health benefit, the promotion and availability of a preparation acceptable for those who avoid dairy-like products should be considered.

Probiotic use was commoner in subjects relatively early in the course of their disease, but this was independent of the age of the patient. It will be interesting to explore if this association is present in larger cohorts: one explanation may be that newly diagnosed patients are keen to explore many avenues to help their disease, yet those with established disease may have adjusted to their disease and have no desire for, nor awareness of, other potential therapies.

Perhaps not surprisingly, probiotic use was associated with a perceived lower quality of life, suggesting that those most disaffected with current disease management are more likely to look elsewhere for alternatives. Interestingly probiotic use was associated with lower scores on the domains measuring the emotional and social domains assessing quality of life relative to the psychosocial impact of the disease but not those measuring the physical effects of the disease. Therefore, this may indicate that those who are more affected by the disease in an emotional or social respect are more likely to seek further means to control or improve their disease. Thus specifically asking about probiotic use may be a useful surrogate marker for detecting those IBD patients with the most dissatisfaction or difficulty with their current situation and may allow more tailored individual interventions.

Despite the ready availability and possible advantages of using probiotics in IBD, there is relatively little data concerning the use of probiotics in this patient group. Several studies have included an examination of probiotic use within the wider sphere of “complementary and alternative therapies”, which obviously has a much wider reach than just probiotics, and certainly it can be hypothesized that probiotics may have a more specific and targeted effect on gastrointestinal health compared with more general well-being than may be seen with other CAMs. Given these possible more direct benefits of probiotics compared to other CAMs, the specific factors associated with probiotic use remain to be determined. One study in IBD outpatients from a Canadian teaching centre showed that overall 56% of patients were using some form of CAM but only about half of this use was probiotics ^13^. Similar to our study, higher educational achievement was associated with CAM use ^13^. Again, problems with, and dissatisfaction with, current medical therapies seemed to be an important driver in CAM use: although in that study probiotic use was higher in those apparently with more active disease (assessed by the Harvey-Bradshaw index), there was no difference in S-IBDQ scores between CAM users and non-users ^13^. A postal study of Canadian IBD patients ^14^ again confirmed that CAM use was common (47% had ever used, with 23% current users). Probiotics, in the form of *Acidophilus species*, were commonly used (19% of patients being current users) but overall herbal and plant therapies were more common (41%) and massage therapies (18%) were almost as common ^14^. Interestingly between the Canadian provinces there were regional variations, with probiotic use being more common in all other provinces (20–25% of patients) compared with Quebec (8%). A further internet-based cross sectional survey of IBD patients (mainly from North America) demonstrated that 34% of patients were current users of at least one type of CAM with vitamins and herbal products the most popular ^15^. The only factors that seemed associated with CAM use in this study were not having had previous IBD-related surgery or not having received steroids ^16^; data for probiotics were not reported separately. Further studies from Germany ^16^ and Hungary ^17^ have again confirmed that CAM use is common in the IBD population but also illustrate that different products predominate in different areas. Probiotics were less commonly used in these populations, despite probiotics being prescribable for IBD in Germany. In Germany homeopathy was most common ^16^, whilst in Hungary herbal tea and homeopathy were predominant, with minimal use of probiotics ^17^. These studies suggest that many other factors are important in determining the choice of any specific CAM and that the factors which relate specifically to probiotic use in different geographical and sociodemographic groups use remain to be determined.

In conclusion, despite the relatively small size of our sample, we have shown that the use of probiotics is common in a UK IBD cohort. Use of probiotics was associated with Crohn’s disease more than ulcerative colitis, relatively short duration of disease since orginal onset, lower perceived quality of life and higher educational attainment. The taste of the probiotic supplements was equally as popular as potential disease-modifying effects as a reason for using probiotics. Non-users were influenced by the costs involved, perceived lack of benefit and a concern that diary-based products might make symptoms worse. Further studies are warranted to determine the different patient- and disease-related factors that influence the use of probiotics and also to determine any positive or negative effects of probiotic use on the behaviour of IBD, complications, concordance with prescribed medication and patient well-being.

## Figures and Tables

**Figure:**
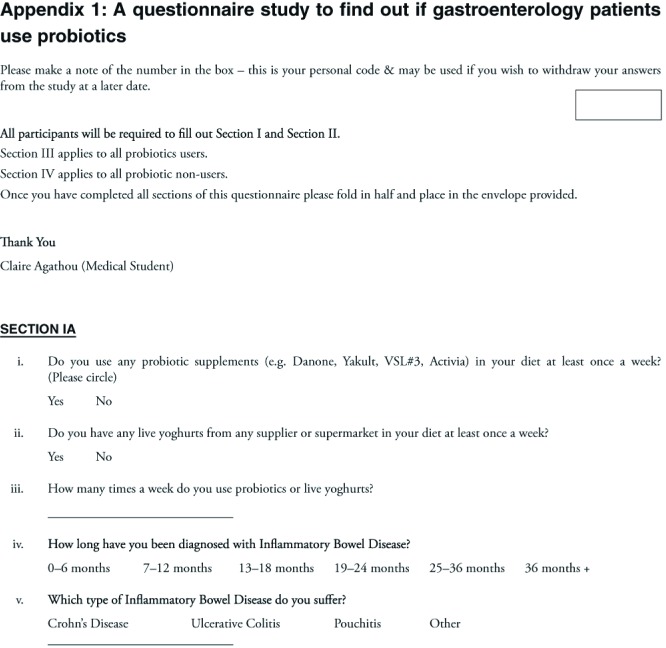


**Figure:**
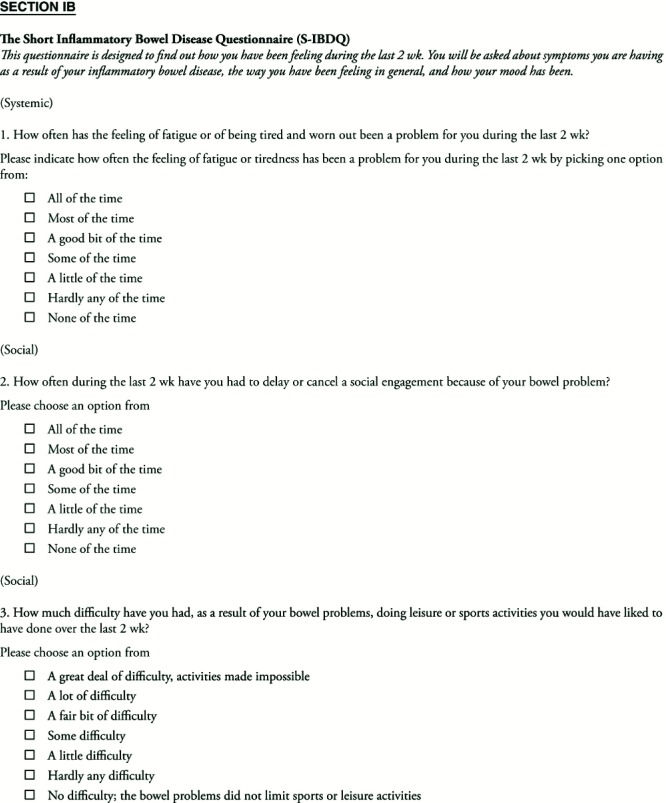


**Figure:**
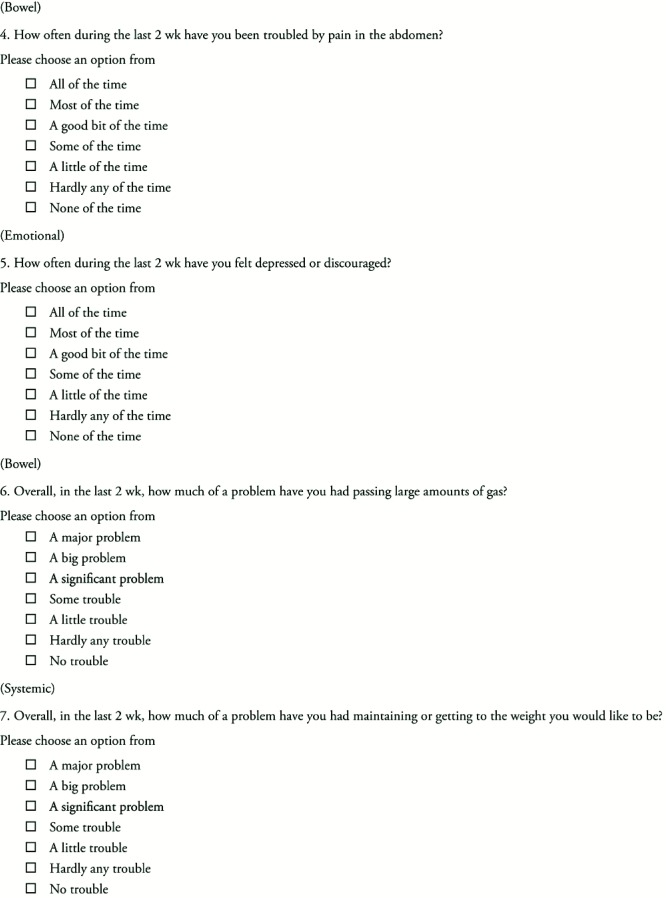


**Figure:**
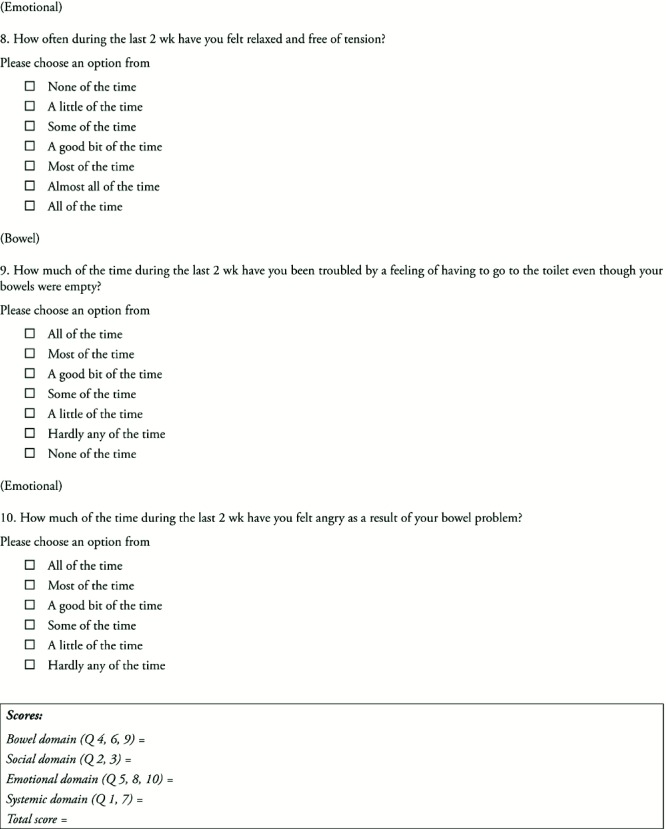


**Figure:**
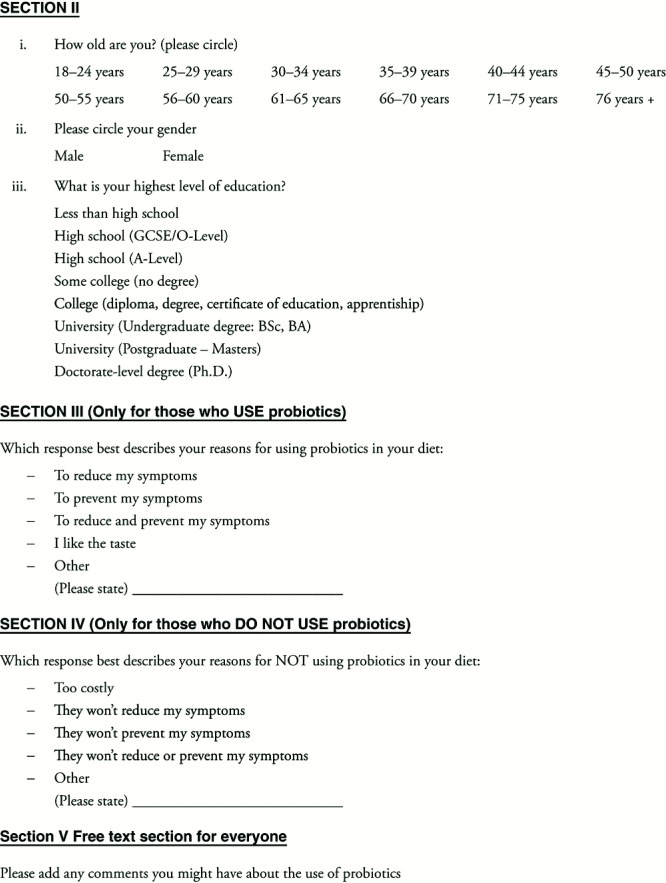


## Notes

Appendix 2: Use of Probiotics in IBD - individual subject level data. Table showing subject-level data for demographics, use and reason for use of probiotics and individual answers to the S-IBDQ questions.
