# Supplementary material for: Factors associated with the use of probiotics in patients with inflammatory bowel disease
Source: F1000Res. 2013 Mar 4;2:69. [Version 1] doi: 10.12688/f1000research.2-69.v1 (PMC3901512; doi:10.12688/f1000research.2-69.v1)
Supplement: Peer review history

# Peer review: Referee response for version 1

**Published:** 2013-05-09

**Authors:** Stephen Lewis

**Affiliations:** 1 Gastroenterology, Derriford Hospital, Plymouth, UK

I enjoyed reading this small study but it is clearly underpowered and has no control group making it difficult to draw meaningful conclusions.

**Abstrac** **t**

Background: The second sentence implies that probiotics have an effect on gastrointestinal function, which contradicts some of the later statements saying that their effects are unsubstantiated. I would tend to favour focusing on the study and not entering into any discussion on the effectiveness of probiotics.

**Introduction** **:**This is far too long and could easily be cut in half. Particularly the first paragraph is repeated elsewhere in the introduction and is largely irrelevant to the study. The grammar generally could be more precise. 

**Materials and methods:**I have significant reservations on the over interpretation of statistics in such a small sample size. In particular where you are drawing conclusions using Fisher exact tests on a sample of three patients some groups, this leaves the results open to speculation even if statistically significant. 

**Results:**When stating a result is statistically significant it is appropriate just to say that the findings are significant, 'statistically' becomes a redundant word. If something is not statistically significant then it is not significant. So to state 'men use probiotics less commonly than women but this was not statistically significant' is incorrect, statistically there is no difference. This is repeated throughout the paper and should be corrected.What would be really interesting is if there was a control group of patients who did not have IBD to see whether their probiotic consumption was similar to those with IBD and whether the same confounding factors occurred. At least some comment should be made to this. 

Given that taste preference was one of the main reasons for using probiotics, this factor should be explored in more detail. In particular some examples of costing would be appropriate. How much more expensive are probiotic yoghurts than normal yoghurts? Would patients have taken the probiotics even if they did not have IBD?

I would also like to see some data on length of probiotic use. Presumably if it were used for a while with no benefit then patients would stop taking it. 

The difference between ulcerative colitis and Crohn’s is interesting and I just wonder if this represents differences in quality of life.

Was there any perception that probiotics actually reduced or improved patient’s quality of life or reduce the symptoms?

**Discussion:**Given that taste preference was one of the major findings this perhaps should be more dominant in a discussion. I guess it also would be linked to marketing side, which is quite powerful. Overall there is a bit of repetition and the text is a bit verbose, it could be edited down a little.

I have read this submission. I believe that I have an appropriate level of expertise to confirm that it is of an acceptable scientific standard, however I have significant reservations, as outlined above.

# Peer review: Referee response for version 1

**Published:** 2013-05-09

**Authors:** Sara McCartney

**Affiliations:** 1 Department of Gastroenterology, University College London Hospitals, London, UK

The article is excellently written, clear and interesting. It addresses some of the issues of widely used but often undocumented and poorly evaluated therapies readily available to patients with IBD. The design and methods are clearly laid out and the discussion was relevant and appropriate.

My reservations relate to:

-  Although this is an observational study some comparator data in an age-matched group without IBD would be helpful.  
- The numbers are small and the age distribution of the study group is older than one might expect with IBD (mode age range 61-65). This may limit generalisation to the wider IBD population.
-  Further information as to which probiotic preparations were taken would be helpful.

Overall, however, I feel this should be approved, particularly as a pilot to a larger study when the issues raised above could be fully addressed.

I have read this submission. I believe that I have an appropriate level of expertise to confirm that it is of an acceptable scientific standard, however I have significant reservations, as outlined above.

# Peer review: Referee response for version 1

**Published:** 2013-03-26

**Authors:** Richard Pollok

**Affiliations:** 1 Gastroenterology, St George's Healthcare NHS Trust, London, UK

This is a small study but well designed and well written. It might have been interesting to learn what type of probiotics were being used, this could be submitted as an appendix. 

The conclusion is reasonable, it is an interesting observation that these products seem to be popular early in diagnosis but interest then drops off.

I have read this submission. I believe that I have an appropriate level of expertise to confirm that it is of an acceptable scientific standard.
